# A longitudinal, multi-level comparative study of quality and safety in European hospitals: the QUASER study protocol

**DOI:** 10.1186/1472-6963-11-285

**Published:** 2011-10-26

**Authors:** Glenn B Robert, Janet E Anderson, Susan J Burnett, Karina Aase, Boel Andersson-Gare, Roland Bal, Johan Calltorp, Francisco Nunes, Anne-Marie Weggelaar, Charles A Vincent, Naomi J Fulop

**Affiliations:** 1 National Nursing Research Unit, King's College London, 57 Waterloo Road, London, SE1 8WA, UK; 2 King's Patient Safety and Service Quality Research Centre, King's College London, 150 Stamford Street, London, SE1 9NH, UK; 3 Centre for Patient Safety & Service Quality, Imperial College, St Mary's Campus, Norfolk Place, London, W2 1PG, UK, UK; 4 Department for Media, Cultural and Social Studies, University of Stavanger, 4036 Stavanger, Norway; 5 The Jönköping Academy for Improvement of Health and Welfare, Box 1026, 551 11 Jönköping, Sweden; 6 Institute of Health Policy & Management, Erasmus University Rotterdam, Postbus 1738, 3000 DR Rotterdam, the Netherlands; 7 The Jönköping Academy for Improvement of Health and Welfare, Box 1026, 551 11 Jönköping, Sweden; 8 Instituto Superior de Ciências do Trabalho e da Empresa (ISCTE), Av.ª das Forças Armadas, 1649-026 Lisboa, Portugal; 9 Institute of Health Policy & Management, Erasmus University Rotterdam, Postbus 1738, 3000 DR Rotterdam, the Netherlands; 10 King's Patient Safety and Service Quality Research Centre, King's College London, 42 Weston Street, London, SE1 3QD, UK

## Abstract

**Background:**

although there is a wealth of information available about quality improvement tools and techniques in healthcare there is little understanding about overcoming the challenges of day-to-day implementation in complex organisations like hospitals. The 'Quality and Safety in Europe by Research' (QUASER) study will investigate how hospitals implement, spread and sustain quality improvement, including the difficulties they face and how they overcome them.

The overall aim of the study is to explore relationships between the organisational and cultural characteristics of hospitals and how these impact on the quality of health care; the findings will be designed to help policy makers, payers and hospital managers understand the factors and processes that enable hospitals in Europe to achieve-and sustain-high quality services for their patients.

**Methods/design:**

in-depth multi-level (macro, meso and micro-system) analysis of healthcare quality policies and practices in 5 European countries, including longitudinal case studies in a purposive sample of 10 hospitals. The project design has three major features:

• a working definition of quality comprising three components: clinical effectiveness, patient safety and patient experience

• a conceptualisation of quality as a human, social, technical and organisational accomplishment

• an emphasis on translational research that is evidence-based and seeks to provide strategic and practical guidance for hospital practitioners and health care policy makers in the European Union.

Throughout the study we will adopt a mixed methods approach, including qualitative (in-depth, narrative-based, ethnographic case studies using interviews, and direct non-participant observation of organisational processes) and quantitative research (secondary analysis of safety and quality data, for example: adverse incident reporting; patient complaints and claims).

**Discussion:**

the protocol is based on the premise that future research, policy and practice need to address the sociology of improvement in equal measure to the science and technique of improvement, or at least expand the discipline of improvement to include these critical organisational and cultural processes. We define the 'organisational and cultural characteristics associated with better quality of care' in a broad sense that encompasses all the features of a hospital that might be hypothesised to impact upon clinical effectiveness, patient safety and/or patient experience.

## Background

There is now a good understanding and knowledge of the types of quality improvement (QI) interventions that are undertaken in healthcare [1] but less understanding of how to increase their effectiveness [2].

Studies on healthcare quality increasingly point to understanding organisational issues in health service delivery as central to explaining variations in care and making progress towards sustained quality improvement. The Institute of Medicine's watershed *To Err is Human *[3] and *Crossing the Quality Chasm *[4] reports specifically identified organisational failings as one of the root causes of poor quality, with the latter devoting an entire chapter to analysing healthcare organisations as complex, adaptive systems and the implications of this perspective for implementing change. As elaborated by others [5-7] this perspective includes recognising the multiple levels of the healthcare system. High-level influences such as policy, payment rules, regulation and accreditation are strongly mediated by dynamics and responses not only at the levels of hospitals, but also the smaller care delivery units within hospitals that deliver services directly to patients.

A rigorous, if relatively small, body of research does exist in the health services literature which specifically attempts to unravel this 'black box' of organisation at the hospital level and its impact on the quality of care [8,9]. This work has focused on identifying hospital predictors of successful implementation of quality improvement, typically using multivariate statistical methods and quasi-experimental data, and has highlighted a number of factors that appear to be associated with successfully implementing change in hospitals [10]. The factors that predict successful quality improvement implementation include leadership support [11], particular dimensions of organisational culture and climate [12,13], and team-based structures and composition [14], as well as investing in the measurement of quality and making quality projects 'do-able' [15,16]. As noted previously [17], there is also an increasing evidence-base relating to the factors that influence how 'improving quality' can be successfully implemented and assimilated into the routine practice of frontline clinical teams. Such work has been heavily influenced by the micro-systems focus in the work of researchers from Dartmouth-Hitchcock in the United States [18,19].

There has been a traditional preference for broad, survey-based research to explore factors associated with successful quality improvement (for example the recent EU-funded MARQuIS project [20] and the work of the European Observatory on Health Systems and Policies). However, as Øvretveit and Staines [21] have pointed out: 'Apart from a few projects, the details of which interventions were actually made are often not presented, and there are few adequate or independent research descriptions of actual implementations of organisational and system wide programs over time'. Given the paucity of in-depth studies to date, it is hardly surprising that the minutiae of quality improvement programs and processes remain largely shrouded in mystery. This is not to argue that large-scale surveys of national policies across large numbers of countries are not important; it is simply to say that without further detailed investigation of the findings of such surveys, health care leaders-whilst being aware of broad trends and directions-will remain uninformed as to the detailed 'how to'-or implementation-of successful quality improvement at the hospital level.

The predominant focus in the majority of studies in healthcare quality has been solely on technical factors that are thought to influence the quality of care (despite the socio-technical systems perspective in which information technology and deeper, cultural processes are studied symmetrically). As a consequence it has been all too easy to forget (or simply fail to acknowledge) the fact that every aspect of care is accomplished through people in their everyday actions and interactions with and for each other-a social process. If quality is viewed in this way issues such as identity, politics, leadership, value systems, organisational 'slack', and learning, can begin to receive the same attention as the technical factors that have dominated the research field to date.

Furthermore, most studies have rarely taken the time to construct theories or explanations for what they observe or find in their analyses [22]. This is particularly true of the organisational and cultural dimensions of quality improvement. Unfortunately the existing evidence-base has also been less adept at shedding light on how factors at different levels of a healthcare system relate to one another, and how in practice hospitals should go about influencing and setting 'key success factors' in motion.

The European Union-funded QUASER study will seek to extend recent research that has addressed these theoretical and methodological issues. Bate, Mendel and Robert [17] undertook a three-year international study that was explicitly designed to help practitioners and researchers understand the factors and processes that enable hospitals in the US and Europe (England and the Netherlands) to achieve-and sustain-high quality services for their patients. This original study took as its starting point that whilst technical factors, such as information systems, do play a major role in accounting for the quality 'gap', organisational and cultural factors are crucial in understanding how quality and safety improvement occurs.

Based on in-depth, multi-level case studies of seven leading hospital, this research found that high-performing hospitals were able to achieve, and then sustain, high levels of quality because they recognised and had been extremely successful in addressing-on an ongoing basis-six common challenges. The six common challenges that were identified from the case studies were:

1. structural-organising, planning and co-ordinating quality efforts

2. political-addressing and dealing with the politics of change surrounding any QI effort

3. cultural-giving 'quality' a shared, collective meaning, value and significance within the organisation

4. educational-creating a learning process that supports improvement

5. emotional-engaging and mobilizing people by linking QI efforts to inner sentiments and deeper commitments and beliefs

6. physical and technological-the designing of physical systems and technological infrastructure that supports and sustains quality efforts

The researchers represented these common challenges by means of a 'codebook' which took the form of a checklist that practitioners can use to identify where the organisational gaps in their local improvement efforts may lie and what they may need to do to address them. Based on the systematic review and coding of the organisational case studies, multiple illustrations of the different types of challenges and solutions were extracted from the individual case study narratives and assigned to the different challenges. In total, the codebook includes 56 such solutions spread across the six challenges, all derived inductively from the organisational cases themselves.

The QUASER study will extend and apply this original research in several important ways:

• by studying a range of hospitals at different stages on their quality 'journeys' (as opposed to just high performing hospitals)

• by explicitly including clinical effectiveness, patient safety and patient experience as key components of what we mean by 'quality' (as opposed to focusing just on service improvement)

• by incorporating available qualitative and quantitative measures of quality into a cross-case, comparative analysis (as opposed to a purely qualitative analysis)

• by including a much broader range of countries (England, the Netherlands, Norway, Portugal and Sweden)

• by providing context-specific guidance to (a) hospitals depending on where they are on their quality journey, and (b) payers and those assessing the quality of hospital care

Finally, given that each of the macro (national healthcare system), meso (hospital) and micro (frontline clinical team) levels, separately and in interaction with each other, affects clinical effectiveness, patient safety and patient experience the QUASER study will retain a particular focus on the dynamics and interactions between these different levels [23-25] as possible key determinants of sustained quality in healthcare.

A favourable ethical opinion for this research study was granted by **NRES Committee South East Coast-Surrey **in April 2011, REC reference: **11/LO/0348**.

## Methods/Design

Organisational case studies are a preferred research method within complex and dynamic contexts where it is difficult to isolate variables or where there are strong interactions between variables [26]. The case study can generate hypotheses from exploratory data which can then be tested in wider samples using different methods, and-particularly relevant to the aims of the QUASER project-they address questions of process as opposed to the input/output model of much quantitative research. Process research is characterised by the dynamic study of behaviour within organisations, focusing on organisational context, activity and actions which unfold over time [27,28]. The comparative design we will adopt will allow pattern recognition across the 10 hospitals in order to generate generic as well as issue-specific learning.

Fieldwork will be undertaken in two hospitals in each of the five EU partner countries (England, the Netherlands, Norway, Portugal and Sweden) making a total of ten hospitals, over a period of 12 months. The countries were chosen as they represent variation in the important aspects of healthcare that we want to capture; for example, differences in the way that healthcare services are funded, and in the way that progress had been made in each country on their 'quality journey'.

**Figure 1:**
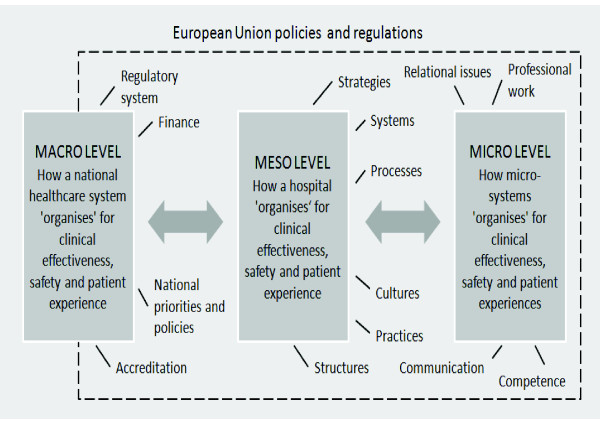
**A Multi-Level Perspective to Study Quality**.

Data collection and analysis will be standardised across all case study sites using an agreed framework. A central feature of the project is to study quality from a multi-level perspective [23-25] incorporating three levels: macro (national healthcare system), meso (hospital) and micro (frontline clinical team) (see Figure 1).

At the meso level all ten hospitals will be studied. At the micro level we will select one hospital in each country and study processes in two clinical micro systems. There will therefore be a total of ten micro system case studies within five hospitals. The rationale for studying two micro systems in a single hospital is to compare the relative influence of macro, meso and micro level processes on quality and to understand how macro and meso level processes are mediated at the micro system level.

### Selection of case study hospitals

A simple and easily communicable selection process has been developed that can be applied in each of the five participating countries to select two hospitals that appear from available indicators to be at different stages of the quality journey, with the final selection also informed by using national accreditation, regulation or similar measures in each country. For our research purposes:

• the hospitals included must be general hospitals, that is, they must include a mix of general medicine and general surgical services, admitting both emergency and planned cases

• the hospitals may be teaching hospitals but in our final selection we will aim to ensure that there are both teaching and non-teaching hospitals

• the hospitals must provide maternity services (as this micro-system will be part of the research in one of the hospitals in each country).

The intention is to select hospitals similar in size; in particular we wish to exclude outliers, for example very large or very small hospitals-in order to be able to recognise the variation that exists between partner countries. The following information about the shortlisted hospitals in each country will therefore also be considered as part of the selection process:

• number of beds

• number of staff

• types of services provided

• type of population served (city, town or rural)

Throughout the selection process we will document the reasons for selecting the process and outcome measures in each country, the sources of data used and the evidence or criteria used to determine the robustness of the data.

### Fieldwork

**Figure 2:**
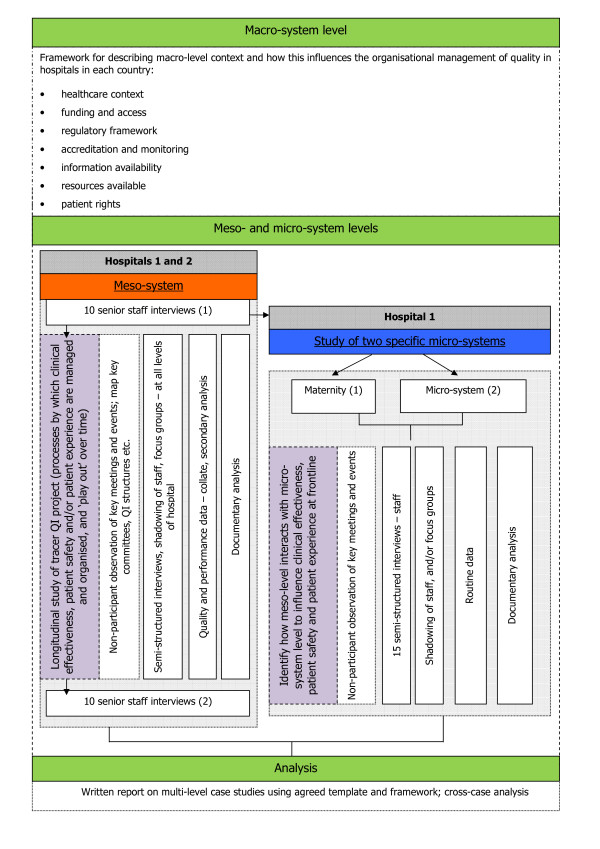
**Protocol for Fieldwork**.

The following fieldwork (see Figure 2) will be undertaken over a 12-month period in each of the five European countries.

#### Macro-system

At the macro level the aim is to understand how broader socio-political influences impact on healthcare quality at the meso and micro level and, in turn, how these are influenced by developments in meso and micro practices. We will draw on existing data in each country. Seven categories of macro level influences on healthcare quality have been identified as shown in Figure 2.

Prior to the fieldwork discussed in detail below each partner country will prepare a structured analysis of the macro-system level, based on these categories, in which the hospital case study sites in their countries are operating.

#### Meso-system

Ten acute hospitals in five EU countries (two from each of the five participating countries) will be invited to participate. In all of the participating hospitals the meso-systems will be studied. At an early stage of engagement with each of the hospitals the national research teams will identify a 'site captain' or contact (usually a member of the senior management team) who will be able and prepared to assist with introducing the research to relevant staff and advising the research team throughout the study.

At the meso-level the aim is to understand organisation-wide structures and processes for managing quality and the complexities of implementing quality improvement programmes. In each of the ten participating hospitals the fieldwork is expected to comprise:

• at least ten semi-structured, audio-recorded interviews with senior/middle managers at the beginning of the fieldwork; staff interviewees will be identified initially through discussions with the 'site captain' in each hospital and then a snowballing approach will be used, as necessary

• observation of key meetings/events throughout the duration of the fieldwork

• the longitudinal study of a 'tracer' quality improvement project or programme (through staff interviews and/or focus groups, non-participant observation of relevant project events/meetings, shadowing of staff and documentary analysis); in one of the two hospitals in each country this 'tracer' project will relate to healthcare acquired infections to enable cross-case analysis between the five countries

• at least ten semi-structured interviews with senior/middle managers at end of fieldwork; these interviews will focus on reflecting back the emerging findings from the fieldwork and seeking the views of the interviewees as to the validity of these.

• using available routinely collected quantitative data indicating hospital quality to assess where the hospital is on its quality journey and help us to understand the relationship between organisational factors and quality.

The semi-structured interview schedule will cover topics such as:

• historical context: the story so far in this hospital with regard to quality

• current quality improvement initiatives

• how quality is organised

• the quality strategy

• supporting frontline staff supported in terms of quality improvement

• measuring quality

• sharing good practice across professional and clinical boundaries

• education, training and development programmes

• external environment and wider networks (regulators, payers).

One practical objective of the meso-system interviews will be to identify the formal organisational structure and systems for managing quality in the hospital. As well as the interviews themselves researchers will also seek to collate any relevant documentation (organisational charts, annual reports, quality strategies, routinely available data on 'quality' etc). From this information the research teams will identify key committees, meetings and events at which to conduct non-participant observation throughout the remaining period of the fieldwork. Fieldnotes will be taken at all meetings that are observed and the research teams will be focusing on providing rich, longitudinal insights into the overall research questions.

Through the meso-system interviews each national research team will also identify a 'tracer' quality improvement project or programme in each of the two hospitals they are studying. In one of the hospitals in each country this tracer project will relate to healthcare acquired infections. The other project may relate to any (or all) of the three components of the QUASER study definition of quality: clinical effectiveness, patient safety and patient experience. The project executive will ensure that-across the ten hospitals-there are exemplars of projects relating to all three components of our definition of quality. The 'tracer' project or programme will ideally be:

• about to start or be in the early stages of implementation

• being implemented in more than one clinical micro-system or service

• have an identified project manager/lead who is able to facilitate research access

• a formal part of the hospitals ongoing quality improvement strategy

• planned to be completed at or after the end of the fieldwork.

The 'tracer' projects will then be studied longitudinally during the remainder of the fieldwork through a combination of semi-structured interview schedules, documentary analysis, focus groups with staff, shadowing of staff and/or non-participant observation of key events/project meetings, with the latter again focusing on providing longitudinal insights into the overall research question. Fieldwork relating to the tracer projects may be undertaken at all levels of the hospitals as appropriate (and may overlap with the fieldwork in the selected microsystems in one of the hospitals in each country). This aspect of the fieldwork (and during the study of the selected micro-systems in one of the hospitals) may include shadowing key staff for agreed periods of time (e.g. a shift). It is not the purpose of the QUASER study to formally evaluate the success or otherwise of the 'tracer' projects but rather to study them-in real time-as exemplars of how quality/quality improvement is implemented to enable lessons to be drawn from across the ten case studies.

The meso-system interviews will also inform the selection of the second clinical micro-system-in addition to maternity services-in one of the hospitals in each partner country. All interviews will be conducted and transcribed in the native language of each partner country. English written summaries will be shared in the group of researchers, to enable the exchange of findings.

### Micro-system

At the micro-system level the aim is to understand the influence of local factors on quality in two clinical micro systems in one hospital in each of the five participating EU countries. It is proposed that maternity care is one of the micro systems selected as it is high on the agenda of quality and safety improvement efforts in all participating countries and therefore data rich. The other clinical micro system will be selected when the maternity case study is underway or completed. The aim is to select a micro system that will contrast with maternity on its 'quality journey' and allow us to study the differing effects of meso level influences at the micro level.

We are particularly interested in how meso level initiatives are implemented at the local level, practitioner's acceptance and perceptions of those initiatives and how such initiatives are shaped and adapted by frontline staff. We are also interested in initiatives that originate at the micro level without prompting from the meso or macro levels and how this occurs. Local leaders and champions of quality and their perceptions of the receptiveness of the meso level of the organisation to quality improvement will be a particular focus of our investigation. The focus will be on the interaction between the macro, meso and micro levels and how processes at either level can either facilitate or hinder quality improvement efforts.

We will be introduced to the leaders (clinical and managerial) of each of the chosen micro-systems by the Phase I 'site captains' (typically a member of the senior management team) we will have worked with in each of the hospitals; the site captain will be asked to assist the research team in arranging initial meetings with micro-system leaders. We will explain the aims and methods of the proposed research to the micro-system leaders, seek to answer any questions they may have about the proposed methods and value of the research, and explain how the findings will be fed back to them and their organisation. As with the meso-system fieldwork we would anticipate each research team conducting 'exit' interviews with leaders of the two micro-systems towards the end of the 12-month period of fieldwork.

Overall, we will spend a minimum of six months working in each of the two microsystems using a multi-method approach; data collection will include a minimum of 15 face-to-face interviews, observations of organisational processes and documentary analysis. Routinely collected quantitative data will be obtained to indicate quality. The semi-structured interview schedule at the micro-system level will cover the following broad topics:

• what it is like to work in this unit/micro-system?

• team and multi-disciplinary working

• culture, 'mindsets' and outlooks

• relationships/collaboration outside unit and hospital

• leaders and leadership styles

• interactions with meso- and macro-systems

• role of information/information technology

• definitions of 'quality'

• the use of guides and guidance

In some of the case studies it may be more appropriate to undertake focus groups with frontline staff; these would largely be structured around the same set of questions as in the face-to-face interviews.

### Identification and recruitment of research participants

Interviewees will be identified through discussions with the site captain but will include at the meso-system level the Chief Executive, Director of Clinical Governance, Director of Nursing, and Director of Operations. Staff members who fulfil key roles in managing quality improvement programmes as well as staff in the selected microsystems will also be included. An email with a covering letter, staff information sheet, and outline of the semi-structured interview schedule to be used will be sent to each potential interviewee at least 2 weeks prior to the date of the proposed interview. Potential interviewees will be asked to confirm their willingness to participate by return email. The staff information sheet and covering letter both make clear that participation is entirely voluntary.

As described above, if appropriate, focus groups with small numbers of frontline staff (up to 8) will be held to supplement or replace the staff interviews at the microsystem level. As with the interviews an email with a covering letter, staff information sheet, and outline of the themes to be discussed at the focus group (as based on the semi-structured interview schedule) will be sent to each potential participant at least 2 weeks prior to the date of the proposed focus group. Potential participants will be asked to confirm their willingness to participate by return email. The staff information sheet and covering letter both make clear that participation is entirely voluntary.

Staff interviewees at the meso and microsystem levels may also be identified through their attendance and participation in events and meetings relating to quality improvement where non-participant ethnographic observation will be undertaken. Additionally, shadowing of staff in appropriate settings depending on the specific microsystem will take place as agreed with the staff member; the presence of the researcher will be renegotiated on a daily basis with both staff and patients. Staff who are invited to agree to be shadowed for an fixed period of time will be identified through nonparticipant observation at meetings/key events using a purposive sampling approach. An email with a covering letter and staff information sheet will be sent to each potential staff member to be shadowed at least 2 weeks prior to the date of the proposed shadowing. Potential staff to be shadowed will be asked to confirm their willingness to participate by return email. The participant information sheet and covering letter both make clear that participation is entirely voluntary.

## Discussion

Building on earlier findings from Bate et al [17], the meso and micro-system fieldwork-as well as the analysis of the macro-system-will seek to answer the following questions:

▪ how is QI structured, planned and co-ordinated? how is quality 'built into' hospitals?

▪ how are the politics of change negotiated?

▪ how are shared understandings & commitment to quality built?

▪ how do staff learn about quality and quality improvement?

▪ how are individual and collective enthusiasm for quality and quality improvement engendered and supported?

▪ how is the physical, informational, social and technological infrastructure used to support quality and quality improvement?

▪ what are the respective roles of the macro-, meso- and micro-system levels in terms of (a) the successful implementation and spread of quality improvement, and (b) sustained quality?

### Data analysis

Our approach to data collection and analysis will be to use a preliminary theoretical framework [29] rather than a purely grounded theory [30], so that data analysis is a combination of induction (data-driven generalisation) and deduction (theory-driven exploration of hypotheses) [27]. This approach has been used previously by the team in organisational research [31]. We want to try to understand at a deep level the kind of processes that enable hospitals in Europe to achieve quality improvement; what is it that enables them to achieve excellence (as perceived by their patients and peers and supported by clinical and performance data), and what enables them to continually improve their services over considerable periods of time.

### Validity and reliability

Regular meetings of the research teams from each of the partner countries will provide opportunities to discuss and refine the protocol during the course of the fieldwork but it is also anticipated that research teams will be in regular contact in the periods between these meetings to share lessons and discuss any problems that may arise. This will ensure that the fieldwork conducted in the different countries is consistent and reliable. Ongoing discussions amongst researchers and a wider advisory board will provide opportunities for reflexivity and the development of insights into the effect of context on quality improvement.

### Generalisability

In addition to the fieldwork described above the development of the outputs arising from the study (a Quality & Safety Guide for Hospitals and a Framework for Assessing Hospital Quality) will be informed by a parallel process of translational workshops involving both hospitals and payers in a wider stakeholder group from a broader range of European countries. These stakeholders will attend three translational workshops during the course of the project ensuring that issues facing hospitals across the EU will be considered and that the eventual outputs of the project have been designed for the needs of users across the EU. The stakeholders will review progress and ensure that the lessons and findings from the research are going to be relevant and of use to them; they will also provide input on how the materials/tools that are ultimately designed may need to be customised for their particular national contexts.

## List of Abbreviations

EU: European Union; QUASER: Quality and Safety in Europe by Research; QI: Quality Improvement.

## Competing interests

The authors declare that they have no competing interests.

## Authors' contributions

GR jointly drafted the original proposal, prepared the meso and micro-system fieldwork protocol, and drafted this paper; JA, NF & SB jointly drafted the original proposal and commented on the meso and micro-system fieldwork protocol (JA also prepared the macro-system framework and parallel stakeholder process that will inform the development of outputs from the project; NF is principal investigator for the study; SB led the hospital selection process); KA, BAG, RB, JC, FN, AM W, & CV all commented on the fieldwork protocol and macro-system framework, and contributed to the design of the hospital selection process to be applied in their respective countries. All authors read and approved the final manuscript.

## Pre-publication history

The pre-publication history for this paper can be accessed here:

http://www.biomedcentral.com/1472-6963/11/285/prepub
